# Endogenous assessment of chronic myocardial infarction with T_1ρ_-mapping in patients

**DOI:** 10.1186/s12968-014-0104-y

**Published:** 2014-12-20

**Authors:** Joep WM van Oorschot, Hamza El Aidi, Sanne J Jansen of Lorkeers, Johannes MIH Gho, Martijn Froeling, Fredy Visser, Steven AJ Chamuleau, Pieter A Doevendans, Peter R Luijten, Tim Leiner, Jaco JM Zwanenburg

**Affiliations:** Department of Radiology, University Medical Center Utrecht, Heidelberglaan 100 3582 CX, Utrecht, The Netherlands; Department of Cardiology, University Medical Center Utrecht, Utrecht, The Netherlands; Philips Healthcare, Best, The Netherlands

**Keywords:** Heart, Fibrosis, Heart failure, Cardiovascular magnetic resonance, Endogenous contrast, T1ρ-mapping, T1-mapping, MOLLI, Spin-lock, Native contrast

## Abstract

**Background:**

Detection of cardiac fibrosis based on endogenous magnetic resonance (MR) characteristics of the myocardium would yield a measurement that can provide quantitative information, is independent of contrast agent concentration, renal function and timing. In *ex vivo* myocardial infarction (MI) tissue, it has been shown that a significantly higher T_1ρ_ is found in the MI region, and studies in animal models of chronic MI showed the first *in vivo* evidence for the ability to detect myocardial fibrosis with native T_1ρ_-mapping. In this study we aimed to translate and validate T_1ρ_-mapping for endogenous detection of chronic MI in patients.

**Methods:**

We first performed a study in a porcine animal model of chronic MI to validate the implementation of T_1ρ_-mapping on a clinical cardiovascular MR scanner and studied the correlation with histology. Subsequently a clinical protocol was developed, to assess the feasibility of scar tissue detection with native T_1ρ_-mapping in patients (n = 21) with chronic MI, and correlated with gold standard late gadolinium enhancement (LGE) CMR. Four T_1ρ_-weighted images were acquired using a spin-lock preparation pulse with varying duration (0, 13, 27, 45 ms) and an amplitude of 750 Hz, and a T_1ρ_-map was calculated. The resulting T_1ρ_-maps and LGE images were scored qualitatively for the presence and extent of myocardial scarring using the 17-segment AHA model.

**Results:**

In the animal model (n = 9) a significantly higher T_1ρ_ relaxation time was found in the infarct region (61 ± 11 ms), compared to healthy remote myocardium (36 ± 4 ms) . In patients a higher T_1ρ_ relaxation time (79 ± 11 ms) was found in the infarct region than in remote myocardium (54 ± 6 ms). Overlap in the scoring of scar tissue on LGE images and T_1ρ_-maps was 74%.

**Conclusion:**

We have shown the feasibility of native T_1ρ_-mapping for detection of infarct area in patients with a chronic myocardial infarction. In the near future, improvements on the T_1ρ_ -mapping sequence could provide a higher sensitivity and specificity. This endogenous method could be an alternative for LGE imaging, and provide additional quantitative information on myocardial tissue characteristics.

## Background

Detection of the location and extent of myocardial scarring is important for the prognosis of patients with myocardial remodeling [1]. Post-infarct formation of myocardial fibrosis can lead to adverse cardiac remodeling and subsequently, to heart failure. The current *in vivo* reference standard for detection of myocardial scar tissue is late gadolinium enhancement (LGE), where the prolonged retention of gadolinium contrast agent (CA) in regions of myocardial fibrosis results in increased signal intensity on T_1_ weighted cardiovascular magnetic resonance (CMR) images. The presence and extent of LGE CMR carries important prognostic value as has been demonstrated in several ischemic and non-ischemic cardiomyopathies [2–4].

LGE is a validated method with a high sensitivity to discriminate infarcted from healthy myocardium. However, to guide and evaluate medical treatment, more information is needed about the heterogeneity of myocardial damage associated with diverse cardiac disease processes, as this damage is the substrate of arrhythmias, and a possible target for CMR-guided arrhythmia ablation. In order to provide this information, a shift from mere visualization to quantification of myocardial fibrosis is needed. Furthermore, a truly noninvasive method based on endogenous contrast without the requirement for an exogenous contrast agent would be preferable, since gadolinium enhanced CMR with some agents is off-label use [5]. Allergic reactions after intravenous administration of gadolinium-based contrast agents are very rare but potentially life threatening [6,7], and the method cannot be applied in patients with severe renal failure [8]. A quantitative method capable of detecting myocardial fibrosis based on endogenous MR characteristics of the myocardium could, therefore, be a valuable tool, complementary to LGE [9].

In the field of orthopedics, the MR relaxation parameter T_1_ in the rotating frame (T_1ρ_) is well established as measure for the collagen content in cartilage [10,11]. Recent studies have hypothesized that this method may be applicable to directly image collagen in the heart, and therefore would be a promising candidate for detection of chronic myocardial infarction [12,13]. The T_1ρ_ relaxation time describes relaxation while the magnetization is in the rotating frame, in the presence of a so-called spin-lock pulse. A spin-lock pulse is a low amplitude radiofrequency (RF) pulse on-resonance with the precessing transverse magnetization. By acquiring images with varying T_1ρ_ weighting, a so-called T_1ρ_-map can be calculated.

In *ex vivo* MI tissue, it has been shown that the T_1ρ_ relaxation time is sensitive to changes in macromolecular content, and that a significantly higher T_1ρ_ is found in the MI region [12]. Studies in animal models of chronic MI showed the first *in vivo* evidence for the ability to detect myocardial fibrosis with T_1ρ_-mapping [14,15]. Furthermore it has been shown that the addition of T_1ρ_ weighting to a conventional gradient echo sequence improves the contrast between acutely infarcted and noninfarcted myocardium in patients in an acute (63 ± 40 hours) myocardial infarction [13]. However, this method has not yet been reported for assessment of chronic MI in humans.

The aim of the current study is to explore the potential of T_1ρ_-mapping to detect fibrosis in patients with chronic ischemic heart disease on a standard clinical MR scanner. Therefore, we first performed a study in a porcine animal model of chronic MI to reproduce initial results in literature and to validate the implementation of the technique. In a second study the method was translated to a clinical protocol, and the feasibility of detecting scar tissue with native T_1ρ_-mapping in patients with chronic MI was assessed and correlated with LGE CMR.

## Methods

### Animal study

#### Animal model

All *in vivo* animal experiments were conducted in accordance with the Guide for the Care and Use of Laboratory Animals prepared by the Institute of Laboratory Animal Resources. Experiments were approved by the Animal Experimentation Committee of the University Medical Center Utrecht.

Nineteen Dalland landrace pigs (69 ± 5 kg) underwent a 90-minute percutaneous balloon occlusion of the midpart of the left anterior descending (LAD) coronary artery, followed by reperfusion. 16 animals survived the LAD occlusion.

#### CMR method: in vivo animal study

Eight weeks after myocardial infarction, an *in vivo* CMR was performed under anesthesia on a clinical 3 T MR scanner (Achieva TX, Software release 3.2.1, Philips Healthcare, Best, The Netherlands), using a commercially available 32-channel cardiac receive coil.

**Figure 1:**
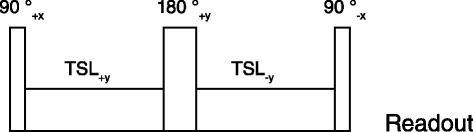
**Spin lock pulse sequence used to obtain T**_**1ρ**_ **weighted images.** The pulse sequence consists of 2 continuous RF pulses with opposite phase to compensate for B_1_ variations, and a refocusing pulse between the spin-locking halves to compensate for B_0_ errors, followed by the readout.

T_1ρ_-mapping was performed using a 3D, T_1ρ_ -prepared, multi-shot gradient echo sequence. T_1ρ_ –preparation was performed using a spin-lock pulse (Figure 1) consisting of 2 continuous RF pulses with opposite phase to compensate for B_1_ variations, and a refocusing pulse between the spin-locking halves to compensate for B_0_ errors [16,17]. The amplitude of the spin-lock pulse was set to 500 Hz (11.7 μT), and five images with different spin-lock (SL) preparation times were acquired (SL = 1, 10, 20, 30, 40 ms). Other parameters were: bandwidth/pixel = 287 Hz, TE/TR = 2.6/5.3 ms, resolution = 1.5 × 1.5 mm^2^, slice thickness = 6 mm, FOV = 336 × 336 mm^2^, flip angle = 8 degrees, 8 TFE shots, number of signals averaged (NSA) =2, shot interval = 2 heart beats.

After the T_1ρ_-mapping 0.2 ml/kg gadobutrol (Gadovist, Bayer Healthcare, Berlin, Germany) was injected and LGE CMR was performed 15 minutes after injection. A look-locker scout sequence was performed to optimize nulling of the remote myocardium. LGE imaging parameters were: TI = 200–250 ms, TE/TR = 1.5/4.7 ms, resolution = 1.5 × 1.5 mm^2^, slice thickness = 6 mm, FOV = 300 × 300 mm^2^, flip angle = 25 degrees, 63 TFE shots).

#### Histology

After the CMR examination the animals were sacrificed, and triphenyltetrazolium chloride (TTC) staining of serially sectioned heart slices was performed (slice thickness = 2 cm). Heart tissue from infarct, borderzone and remote areas were fixated in formalin, embedded in parafine, and 5 μm slides were stained with picrosirius red for fibrosis quantification.

#### CMR methods: Ex vivo animal study

From 5 pigs the third sectioned slice from the apex, was scanned before histology on a clinical 1.5 T MR scanner (Achieva, Philips Healthcare, Best, the Netherlands) in an 8 channel head coil. T_1ρ_ dispersion was measured using a 3D, T_1ρ_ -prepared, multi-shot gradient echo sequence. A T_1ρ_ map was calculated by acquiring five images with different spin-lock preparation time (SL = 1, 10, 20, 30, 40 ms). The amplitude (B_1_) of the spin-lock pulse was varied, to assess the T_1ρ_ dispersion (B_1_ = 0, 100, 200, 500, 750, 925 Hz). The maximum B_1_ of the spin-lock pulse was limited by the maximum B_1_ achievable by the transmit coil. Other imaging parameters were: bandwidth/pixel = 889 Hz, TE/TR = 1.7/3.9 ms, resolution = 1 × 1 mm^2^, slice thickness = 1 mm, 25 slices, FOV = 180 × 180 mm^2^, flip angle = 8 degrees, 64 TFE shots, NSA =1 and shot interval = 3000 ms.

### Patient study

#### Patients

Twenty-one patients with a first reperfused ST-segment elevation MI underwent a CMR between 3 and 12 months after the acute event (Table 1). Written informed consent was obtained from all participants, and the study was approved by the local Ethical Review Board of the University Medical Center Utrecht. Five healthy young control subjects (5 male, age 25 ± 3 years) were scanned to confirm measurement of the remote tissue.

**Table 1:** **Patient characteristics**

	**Patients with MI (n = 21)**
Sex	19 male, 2 female
Age, years	55.4 ± 8.7
BMI	27.0 ± 3.2
Smoking	n = 6; 28.6%
Hypertension	n = 10; 47.6%
Diabetes	n = 3; 14.3%
Hyperlipidemia	n = 13; 65%
Family history of vascular disease	n = 5; 23.8%
Antihypertensive drugs	n = 20; 95.2% (70% β-blockers, 25% Diuretics, 5% ACE-inhibitors)
Statin use	n = 20; 95.2%
Antithrombotic therapy	n = 21; 100%
Age infarct, days	263 ± 139
Lesion	n = 10; 47.6% LAD, n = 10; 47.6% RCA, n = 1; 4.8% LCX
LV ejection fraction	55.7 ± 7.4%

## CMR methods

All subjects were imaged on a clinical 1.5 T MR scanner (Achieva, Philips Healthcare, Best, the Netherlands), using a 5-channel cardiac receive coil. T_1ρ_-mapping was performed using a 2D T_1ρ_-prepared balanced steady-state free precession (SSFP) gradient echo sequence, using the same spin-lock preparation scheme as in the animal study (Figure 1). In the first three patients a multi-shot gradient echo readout instead of a balanced SSFP readout was used, due to further optimization of the protocol. The amplitude of the spin-lock pulse was set to 750 Hz (17.6 μT), and four images with different spin-lock preparation times were acquired (SL = 1, 13, 27, 45 ms). Other parameters were: bandwidth/pixel = 530 Hz, TE/TR = 1.94/3.9 ms, resolution = 1.5 × 1.65 mm, slice thickness = 6 mm, slice gap = 4 mm, FOV = 288 × 288 mm^2^, flip angle = 50 degrees, 2 TFE shots, NSA = 2, SENSE acceleration = 1.5, shot interval = 3 heart beats. In all participants, the T_1ρ_-mapping was performed in 8 short axis slices, acquired in late diastole during expiration breath holds, covering the heart from apex to base.

In the patients, LGE CMR was performed in the same 8 short axis slices, 15 minutes after intravenous contrast injection (0.2 ml/kg contrast agent (Gadovist, Bayer Healthcare). A look-locker scout was performed to optimize nulling of the remote myocardium. LGE imaging parameters were: TI = 300–340 ms, TE/TR = 3.5/7.1 ms, resolution = 1.5 × 1.65 mm^2^, slice thickness = 8 mm, slice gap = 4 mm, FOV = 288 × 288 mm^2^, flip angle = 25 degrees, 5 shots).

### Post processing and image analysis

For both the animal and the patient studies, T_1ρ_-maps were calculated by pixelwise fitting of a mono-exponential decay function in Matlab (Release 2012a, Mathworks, Massachusetts, United States).

The LGE images and T_1ρ_ maps were scored by a radiologist (TL) with over 10 years experience in the evaluation of CMR. Images were scored for image quality, location of the infarct, and transmurality.

The LGE images and T_1ρ_ maps were scored separately using the 17 segments AHA-model [18]. The T_1ρ_-maps were scored two times by the observer. The first time the observer was untrained, and blinded for clinical characteristics, results of conventional LGE imaging and the LGE images were not shown. The second time the observer was trained for looking at T_1ρ_-maps, and the LGE images were shown along with the T_1ρ_-maps, but the observer was blinded for clinical characteristics and the results on the scoring of the LGE images.

Segmentation of infarct and remote myocardium was based on the 2SD segmentation method on the LGE images, and this mask was then applied on the corresponding T_1ρ_-maps to calculate T_1ρ_-values for infarct and remote myocardium [19].

### Statistics

Statistical analysis was performed with GraphPad Prism (GraphPad Software, California, United States). Group comparison was performed using a two-way ANOVA analysis, and considered significant at p < 0.05.

## Results

### Animal study

In 9 out of 16 animals, image quality of the images was sufficient to calculate a T_1ρ_ map. In the other animals, acquisition problems (trigger problems, n = 1) and artifacts (field inhomogeneities (n = 3), foldover-artifacts (n = 2), and motion artifacts due to breathing (n = 1)) led to insufficient image quality to calculate a map.

**Figure 2:**
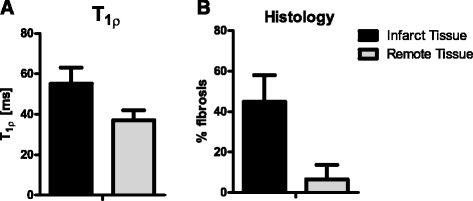
***In vivo*** **T**_**1ρ**_ **relaxation time versus** ***ex vivo*** **histology.** **A:** T_1ρ_ relaxation time measured *in vivo* in a porcine animal model is significantly higher in infarct area (57 ± 11 ms) compared to healthy myocardium (37 ± 4 ms) (p < 0.001) **B:** The amount of fibrosis in the infarct area (44.9 ± 13.2%) was also significantly higher compared to the remote myocardium (6.6 ± 7.1%) (p < 0.0001).

**Figure 3:**
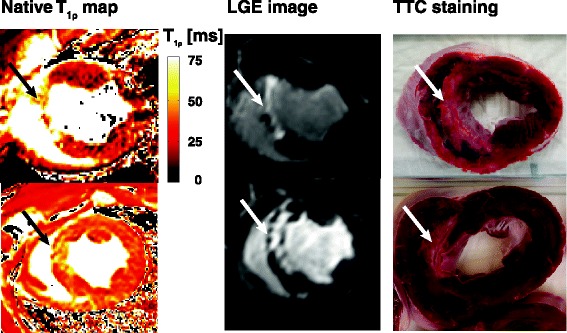
**Short axis** ***in vivo*** **T**_**1ρ**_ **-maps of two different animals with corresponding LGE images and** ***ex vivo*** **TTC staining in a porcine animal model 8 weeks after MI.** In the top T_1ρ_-map artefacts can be observed in the left ventricular free wall, which are likely caused by the effect of B_0_ and B_1_ inhomogeneities on the spin-lock pulse.

Mean ejection fraction in the animals 8 weeks after MI was 39.4 ± 8.1%. The T_1ρ_ relaxation time was significantly higher in the infarcted region (57 ± 11 ms), compared to healthy remote myocardium (37 ± 4 ms) (p < 0.0001) (Figure 2A). Histology showed that the amount of fibrosis in the infarct area (44.9 ± 13.2%) was also significantly higher compared to the remote myocardium (6.6 ± 7.1%) (p < 0.0001) (Figure 2B). (see Figure 3).

**Figure 4:**
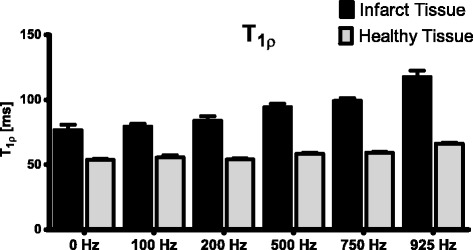
**T**_**1ρ**_ **dispersion measured in** ***ex vivo*** **porcine hearts (n = 5) 8 weeks after MI.** Errorbars indicate standard deviation.

*Ex vivo* scans of the sectioned heart slices showed that the T_1ρ_ contrast between healthy and infarct myocardium increases with an increasing spin lock amplitude (Figure 4).

**Figure 5:**
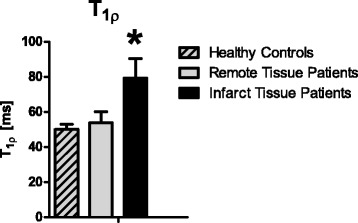
**T**_**1ρ**_ **relaxation time measured in patients with chronic MI is significantly higher in the infarct area (79 ± 11 ms) compared to healthy myocardium (54 ± 6 ms) (p < 0.0005), and compared to myocardium in healthy young volunteers (50 ± 3 ms) (p < 0.0005).**

**Figure 6:**
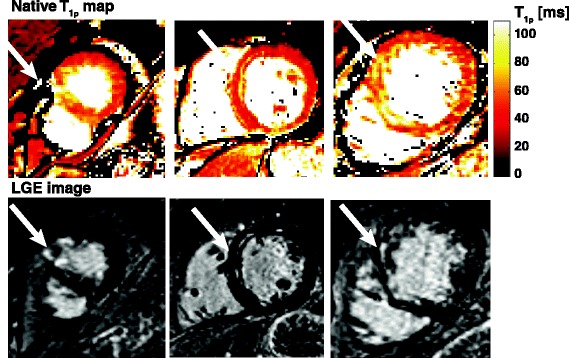
**Short axis T**_**1ρ**_ **-maps with corresponding LGE images in 3 different patients.** Arrows indicate the infarcted area.

### Patient study

The patient characteristics of the 21 patients are listed in Table 1. Mean ejection fraction in the patients after MI was 55.7 ± 7.4% (Table 1). Image quality of the T_1ρ_-maps was good in 9 out of 21 patients, acceptable in 6 patients, and poor in 6 patients. Most important reasons for reduced image quality of the T_1ρ_-maps were misregistration of the different T_1ρ_-weighted images, due to a difference in breath hold position for the scans, and fold-over artefacts.

The T_1ρ_ relaxation time was significantly higher in the infarct region (79 ± 11 ms), compared to healthy remote myocardium (54 ± 6 ms), p < 0.0005 (Figure 5). In the healthy young control participants the T_1ρ_ relaxation time was significantly lower compared to the infarct region (50 ± 3 ms), p < 0.0005. The difference between remote myocardium in patients and myocardium in the healthy young control participants was not significant (p = 0.24).

On both LGE and T_1ρ_ 357 segments were scored, of which 31 segments could not be assessed, due to registration artefacts.

The results of the double blinded untrained scoring are listed in Table 2a. In 93 segments enhancement was scored on a LGE image, and in 63 segments on the T_1ρ_ map. Overall overlap on LGE images and T_1ρ_-maps was 72%.

**Table 2:** **Score LGE versus T**_**1ρ**_ **in patients with chronic MI (n = 21), using the 17 segments AHA-model**

**a:**			
	LGE positive	LGE negative	
T_1ρ_ positive	32	31	0.51 (positive predictive value)
T_1ρ_ negative	61	202	0.77 (negative predictive value)
	0.34 (sensitivity)	0.87 (specificity)	
**b:**			
	LGE positive	LGE negative	
T_1ρ_ positive	71	63	0.53 (positive predictive value)
T_1ρ_ negative	22	170	0.89 (negative predictive value)
	0.76 (sensitivity)	0.73 (specificity)	

a: Scoring was performed double-blinded and the radiologist was not trained to look at T_1ρ_ maps.

b: Scoring of the T_1ρ_ maps was performed unblinded with the T_1ρ_ map next to the LGE image, and the radiologist was trained to look at the T_1ρ_ maps.

Results of the unblinded scoring after training can be found in Table 2b. A significant increase in sensitivity was found (0.76 vs. 0.34). In 93 segments enhancement was scored on a LGE image, and in 134 segments on the T_1ρ_ map. Overall overlap on LGE images and T_1ρ_-maps was 74%.

## Discussion

To our knowledge, this is the first report of *in vivo* detection of chronic myocardial infarction in patients using native T_1ρ_ –mapping without the use of gadolinium contrast agents. Areas of myocardial fibrosis as identified with this approach corresponded reasonably well with conventional LGE images (Figure 6). While the results with the current implementation are promising, sensitivity and specificity is lower compared to the LGE method. We expect that improvements to the implementation will solve this issue, as discussed below. Since T_1ρ_ -mapping requires no contrast agent, it provides a truly noninvasive method, and therefore has the potential to become an alternative to the LGE method in patients with severe renal failure who are unable to receive a contrast agent.

In the animal model we found a significantly higher T_1ρ_ relaxation in the infarct region, compared to healthy myocardium (Figure 2). Histology showed that the collagen fraction in this area was higher compared to macroscopically normal remote myocardium. These results are in accordance with previous findings in other animal studies [12,15]. Similar to the findings in the animal model, in patients with a chronic myocardial infarction we also found a significant higher T_1ρ_ value in the infarct area. The T_1ρ_ relaxation times found in patients were higher than in the animal model, which could be caused by multiple factors. It is known that the T_1ρ_ relaxation time depends on the strength of the main B_0_ field, and decreases with a higher B_0_ [20]. Also, the higher amplitude for the spin-lock B_1_ pulse in the patient study leads to higher values for T_1ρ_, as we also found in the T_1ρ_ dispersion results (Figure 4). Finally we used an increased trigger interval of 3 beats in the patient study, which enabled a better estimation of the true T_1ρ_ value because of reduced T_1_ weighting due to incomplete relaxation of the magnetization prior to the next acquisition.

The *ex vivo* T_1ρ_ dispersion results showed that the T_1ρ_ contrast between healthy myocardium and scar tissue increases with a higher B1 amplitude of the spin-lock pulse (Figure 4). This implies that for T_1ρ_ –mapping we should aim for the highest possible B_1_ amplitude. However, on a clinical MR scanner the maximum B_1_ amplitude is limited by the specific absorption rate (SAR) and the performance of the hardware (transmit coil and RF amplifiers). To stay within human SAR limits, a spin-lock amplitude of 500 Hz (11.7 μT) was used at a field strength of 3 T in the animal experiment. The patient study was performed on a 1.5 T system, enabling a higher B_1_ amplitude for the spin-lock pulse of 750 Hz (17.6 μT), because of lower SAR values and a higher B_1_ field available. Another reason to perform the patient study on a 1.5 T system, is that artefacts caused by B_1_ and B_0_ inhomogeneities, as can be seen in Figure 3, are reduced on a lower field strength. The dispersion data suggests that an even higher B_1_ amplitude for the spin-lock pulse would generate more contrast between healthy and fibrotic myocardium. New CMR contrast developments such as relaxation along a fictitious field (RAFF) may have the potential to generate more contrast between infarct and remote tissue for a given B1 amplitude and SAR level [21].

Double-blinded qualitative scoring of the LGE images and T_1ρ_ maps in patients was performed to investigate if the native T_1ρ_ maps can be used to assess the presence and location of myocardial scar accurately. We found a 72% agreement between both methods in the patients (Table 2a). However compared with *in vivo* gold standard LGE imaging, the sensitivity of T_1ρ_ –mapping to detect scar tissue was found to be lower using the present implementation.

The most important reason for the lower sensitivity is that the contrast to noise ratio between healthy myocardium and scar tissue is much higher in LGE imaging. Although the difference in native T_1ρ_ between healthy and infarct tissue is significant, especially smaller infarcts might be more difficult to detect with this method. The infarct size of the patients in this study was small, which can also be concluded from the mean LV ejection fraction of 55.7 ± 7.4% [22].

Important to keep in mind is that the underlying principle to discriminate myocardial scar tissue from normal myocardium with LGE imaging and T_1ρ_ mapping is different. In LGE imaging the difference in contrast agent washout between normal and diseased myocardium is used to identify scar tissue, which reflects changes in perfusion and extracellular volume in the scar area [23]. On the other hand, native T_1ρ_ mapping directly measures the effect of tissue damage and scar tissue formation on the T_1ρ_ relaxation time. T_1ρ_ is known to be sensitive to changes in macromolecular content, and the histology results show that in the infarct region both a significant increase in T_1ρ_ time and fibrosis percentage is found. It is unknown, however, if the increase in T_1ρ_ directly reflects an increase of collagen in scar tissue, or that other changes in tissue composition after MI are involved. Studies in cartilage and protein solutions suggest that other factors such as cellular content and exchange might be involved, since in agarose gels an increase in macromolecule content leads to a decrease in T_1ρ_ time, which is in the opposite direction of our findings [24,25]. Further research should be performed on the mechanism and relation between myocardial fibrosis formation and the myocardial T_1ρ_ relaxation time.

Another reason for the higher sensitivity of LGE, can be that the radiologist is trained to assess myocardial scar on LGE images, but has no experience in looking at T_1ρ_ maps. After the T_1ρ_ maps were scored double blinded, the radiologist was trained to look at T_1ρ_ maps, and scored again with the LGE images shown alongside. This resulted in a significant increase of sensitivity for the detection of infarct area with T_1ρ_ mapping compared to LGE imaging. We also observed that endocardial infarcts were more difficult to detect on T_1ρ_ maps, since partial volume effects, combined with the high T_1ρ_ relaxation time of the blood, make it difficult to distinguish the transition from myocardium to blood on a T_1ρ_ map. Furthermore, since we used multiple breath holds to calculate a T_1ρ_ map, misregistration between the different breath hold positions leads to problems with the calculation of the T_1ρ_ map at the edges of the myocardium.

Future work should aim to overcome these limitations that reduced image quality and assessability of the T_1ρ_ maps in our study. Most important step is the development of a single breath hold T_1ρ_ mapping sequence to obtain a high quality T_1ρ_ map, without registration errors due to multiple breath hold acquisitions. This requires faster cardiac T_1ρ_ mapping sequences, using acceleration methods. Furthermore, a black-blood readout will enable the detection of an endocardial infarct close to the blood, by reducing partial volume effects.

One of the main drawbacks of LGE imaging is the lack of the ability to measure myocardial fibrosis quantitatively. Currently there is a lot of interest in quantitative imaging of myocardial tissue to overcome these limitations, by measuring quantitative contrast enhanced T_1_-maps and extracellular volume (ECV)-maps [26,27]. We believe that native T_1ρ_ –mapping could provide additional quantitative information on myocardial fibrosis in cardiomyopathies, also in patients with diffuse interstitial myocardial fibrosis. Native T_1ρ_ –mapping requires no separate pre- and post-contrast scan, no hematocrit measurement, and is therefore easier to incorporate in a clinical protocol, compared to ECV-mapping. Here we have shown the first evidence that T_1ρ_ mapping can provide quantitative information on myocardial fibrosis in patients with a chronic myocardial infarction. Though speculative, the finding that the remote myocardium of the patients was about 1 SD above the T_1ρ_ value of the healthy subjects, may suggest a slight increase in diffuse interstitial fibrosis in this area, which could become significant if a larger group with more statistical power would be studied. Future work should focus on further validation of the relation between myocardial fibrosis and T_1ρ_, and on the relation with native and contrast-enhanced T_1_ and ECV-mapping.

## Conclusion

In conclusion, we have demonstrated the feasibility of T_1ρ_ mapping for infarct detection, without the use of an exogenous contrast agent, in patients with a chronic myocardial infarction. Although the sensitivity of T_1ρ_ mapping is lower than LGE imaging, there is room for improvements on the T_1ρ_ mapping sequence that could provide a higher sensitivity and specificity. We believe that T_1ρ_ mapping could provide additional information on myocardial tissue characteristics, and be used in the clinic along with quantitative T_1_, T_2_ and ECV mapping methods, to further understand the development of ischemic and non-ischemic cardiomyopathies.

## Abbreviations

MI: Myocardial infarction
LGE: Late gadolinium enhancement
CA: Contrast agent
CMR: Cardiovascular Magnetic Resonance
LAD: Left anterior descending
NSA: Number of signals averaged
SL: Spin lock
FOV: Field of view
SSFP: Steady-state free precession
TE: Echo time
TR: Repetition time
